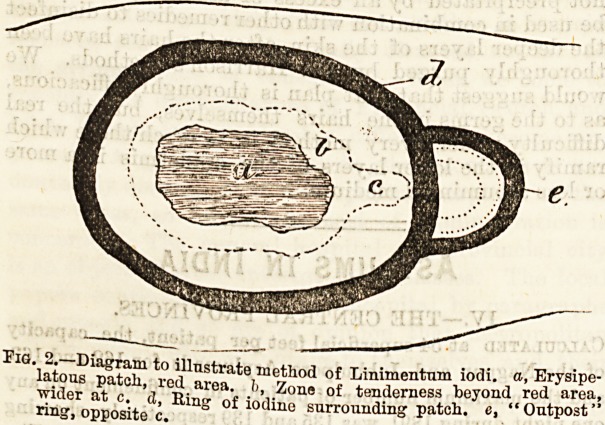# The Treatment of Erysipelas

**Published:** 1893-10-21

**Authors:** 


### ROYAL INFIRMARY, EDINBURGH.

### The Treatment of Erysipelas.

Fortunately erysipelas is not now the scourge of the surgical wards it was wont to be in pre-Listerian times. It is not only less common nowadays, but, judging from the older text-book descriptions, it is a much less formidable disease than it was formerly. Not yet, however, have we been able completely to eradicate it, and every now and again a case appears even in wards where asepsis is the rule, and unless great care is observed an epidemic may ensue. How those sporadic cases originate is not always easy to determine. Sometimes the patient has already been infected before entering the ward, and has brought the disease with him. In other cases it is traced to visitors, out-patients, and fresh admissions to the ward, on more or less reliable grounds.

*Prophyllaxis.*—However difficult it may be to detect the starting point, it is comparatively easy to prevent its spread, if the condition be early recognized. Undoubtedly, when convenient, an erysipelas patient should be isolated to avoid risk of direct or atmospheric dissemination of the disease, as well as to save extra trouble and inconvenience in nursing.

In this hospital, immediately a case of erysipelas is detected it is removed to the Isolation Wards, a building apart from the rest of the hospital, and under the care of a special surgeon and staff of nurses. The bed and bedding are also removed from the ward, the former washed with strong carbolic, and the latter thoroughly sterilized by heat before being again used. No special treatment is adopted in the general wards, that being entirely in the hands of the surgeon to the isolation wards.

When the attack has passed off, and after the temperature has been normal for a day or two, the patient is removed to another ward, where he remains in quarantine for ten days before being readmitted to the ward from which he was originally sent. While here he is attended by a dresser and nurses who are not in contact with other surgical cases.

*Symptoms.*—The onset of the disease is usually marked by a feeling of malaise; often a rigor, or at least a chilliness; not unfrequently headache, nausea and vomiting, and always a rise of temperature to 102 deg. to 105 deg. F., or even higher. The pulse is rapid and weak; there is often albuminuria, and occasionally delirium at night, especially when the disease attacks the head, although this symptom is less common than the books would indicate.

Of the general symptoms, the rapid rise of temperature is the most constant and the most reliable. This temperature remains up as long as the disease continues to spread, and only falls when the inflammation subsides. A fresh outbreak is invariably signalised by a renewal of the pyrexia.

Fig. 1.—Diagram of a Patch of Erysipelas. *a*, Healthy skin beyond patch. *b*, Outside zone in which active changes take place—not red; lymphatics dilated and filled with micrococci. *c*, Bright red area; dilated blood vessels, escape of leucocytes which attack micrococci. *d*, Redness fading, vessels less dilated, macrophages devouring leucocytes and micrococci. *e*, Skin almost normal again.

Locally, the bright rose or brick-red coloration, starting almost invariably in an abrasion of the skin; the definitely circumscribed nature of this, and of the swelling which accompanies it, and the burning pain and excessive tenderness of the part, characterise the disease. It will almost always be found that the area of tenderness on slight pressure exceeds the discoloration by about half an inch all round—a point of pathological, as well as practical importance.

*Treatment.*—The general hygienic conditions of the patient must be satisfactory, a plentiful supply of fresh air being as imperatively demanded as the avoidance of draughts. A mercurial or saline purge is administered at once, and the bowels kept regularly moved during the course of the disease. Fluid food in the shape of beef-tea, mutton broth, milk and potash is given according to the taste of the patient. A stimulant is in almost every case indicated, and may be given in the form of ammonium carbonate, brandy, or whisky, supplemented, if necessary, by a few minims of tincture of strophanthus every three or four hours.

To retail all the methods which are or have been employed in the local treatment of erysipelas would occupy much space without any compensating practical advantage.

While in charge of the isolation wards, Mr. Alexander Miles made some observations on a method of treating the disease based on a consideration of its pathology*. An examination of a patch of erysipelas (Fig. 1) shows that the active inflammatory processes go on in a zone outside the red area (*b*), and that the hyperæmia is rather a result of the disease than its essence—that it is, in fact, nature's attempt to destroy the micro-cocci which are the cause of the inflammation. The suggestion was to produce a zone of hyperæmia in front of the advancing organisms, and so, as it were, anticipate nature's cure. Various

Fig. 2.—Diagram to illustrate method of Linimentum iodi. *a*, Erysipelatous patch, red area. *b*, Zone of tenderness beyond red area, wider at *c*. *d*, Ring of iodine surrounding patch. *e*, "Outpost" ring, opposite *c*.

counter-irritants were tried for this purpose—silver nitrate, tincture of iodine, oil of mustard, &c., but, after a large series of experiments, the pharmacopœal liniment of iodine was found the most satisfactory.

There is nothing new in applying a counter-irritant in front of a spreading patch of erysipelas; it is an ancient practice, but it was done empirically, with the result that the irritant was either too weak to produce hyperæmia (*e.g.*, tincture of iodine), or so strong as to destroy the part and so render the tissues a ready prey to the organisms (*e.g.*, silver nitrate), or the application was made close to the red area, and therefore behind the organisms. From one or other of these causes the application failed, and the method fell into disrepute. The method employed by Mr. Miles is to paint around the erysipelatous area a ring of linimentum iodi about half an inch wide (Fig. 2 *d*). This ring is applied about one inch from the margin of the affected skin, the limit of disease being reckoned by the tenderness on slight pressure, which is usually found to be well in front of the red area (*b*). The diseased skin is marked off with a dermograph pencil to facilitate accurate observations as to spread, and several coats of iodine are painted on, one after the other dries. When any doubt exists as to whether the limit of the disease has been reached a second ring, for convenience called an "out-post," (*e*) is applied about an inch in front of the first. It has been found safest to repeat the painting for two or three days, even although it has never been crossed by the erysipelas. After the iodine dries the part is covered with cotton wool and bandaged.

In all 60 cases were treated by this method. In 24 of these there was no spread beyond a single ring of iodine. In 7 others the ring was overstepped for a very short distance, and then the spread ceased without further treatment. In 14 cases a second ring arrested the onward progress of the disease which had passed the first ring. In 5 cases the iodine entirely failed to stop the erysipelas; and 3 patients died before the treatment had had time to be fairly tested.

## Figures and Tables

**Fig. 1. f1:**
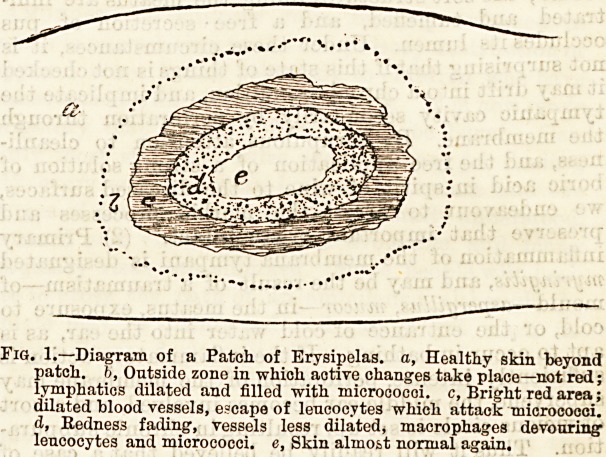


**Fig. 2. f2:**